# Supplementary material for: Identification of photoperiod-induced specific miRNAs in the adrenal glands of Sunite sheep (Ovis aries)
Source: Front Vet Sci. 2022 Jul 22;9:888207. doi: 10.3389/fvets.2022.888207 (PMC9354845; doi:10.3389/fvets.2022.888207)
Supplement: Supplementary file 1 [file Data_Sheet_1.ZIP › Supplementary file/miRDeep2 file.zip!/miRDeep2 file/Novel_Structure/SPLP42A2/Novel_534.pdf]

Provisional ID	:	NC_019477.2_35194
Score total	:	5.4
Score for star read(s)	:	3.9
Score for read counts	:	-1.8
Score for mfe	:	1.8
Score for randfold	:	1.6
Score for cons. seed	:	
Total read count	:	8
Mature read count	:	3
Loop read count	:	0
Star read count	:	5

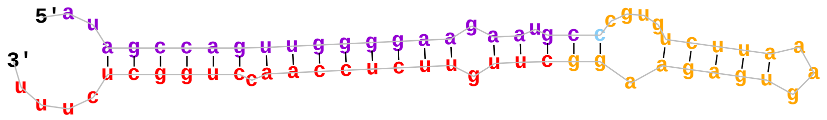

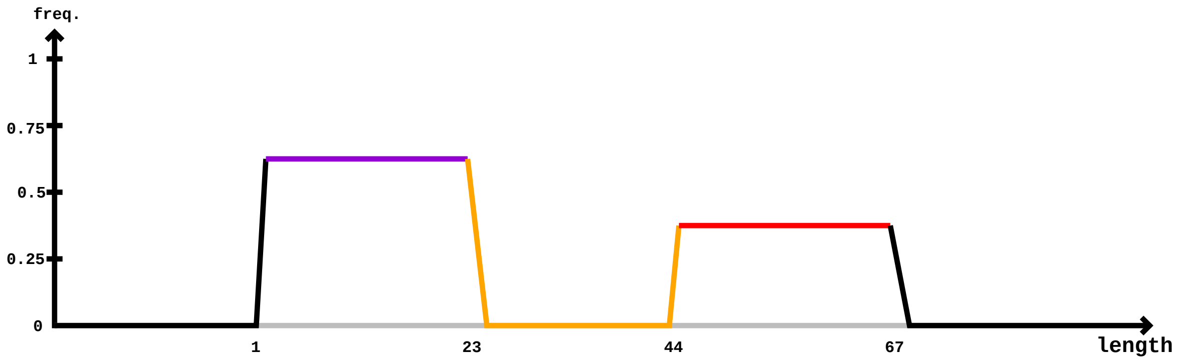

Star

Mature

```
5'- aaggcuaauccuauugaggcauagccaguuggggaagaaugcccgugucuuaaagugagaaggcuuguucuccaaccuggcucuuuauggcugcuggggaaaggcaugaagc   -3'    obs
    aaggcuaauccuauugaggcauagccaguuggggaagaaugcccgugucuuaaagugagaaggcuuguucuccaaccuggcucuuuauggcugcuggggaaaggcaugaagc          exp
    ...(((..((((....((.(((((((((((((((((.((.(((....(((((...))))).))))).)))))))).))))))....))).))....))))..))).......   reads      mm         sample
    ....................auagccaguuggggaagaaugc......................................................................   3          0          seq
    ....................auagccaguuggggaagaaugcc.....................................................................   2          0          seq
    ...............................................................cuuguucuccaaccuggcucuuu..........................   3          0          seq
```
